# Gut microbiota and intestinal barrier function in subjects with cognitive impairments: a cross-sectional study

**DOI:** 10.3389/fnagi.2023.1174599

**Published:** 2023-06-07

**Authors:** Ying Pei, Yan Lu, HuiZi Li, ChengYing Jiang, Lei Wang

**Affiliations:** ^1^Postgraduate Union Training Base of Jinzhou Medical University, PLA Rocket Force Characteristic Medical Center, Beijing, China; ^2^Department of Neurology, PLA Rocket Force Characteristic Medical Center, Beijing, China; ^3^Department of Nutrition, PLA Rocket Force Characteristic Medical Center, Beijing, China; ^4^Institute of Microbiology, Chinese Academy of Sciences, Beijing, China

**Keywords:** gut microbiota, intestinal barrier, cognitive impairment, gut-brain axis, Alzheimer’s disease

## Abstract

**Background:**

Gut-brain axis might play an important role in cognitive impairments by various diseases including Alzheimer’s disease (AD).

**Objective:**

To investigate the differences in gut microbial composition, intestinal barrier function, and systemic inflammation in patients with AD or mild cognitive impairment (MCI), and normal control (NC) cases.

**Methods:**

A total of 118 subjects (45 AD, 38 MCI, and 35 NC) were recruited. Cognitive function was assessed using Mini-Mental State Examination (MMSE), and Montreal Cognitive Assessment Scale (MoCA). Functional ability was assessed using Activity of Daily Living Scale (ADL). The composition of gut microbiome was examined by 16S rRNA high-throughput sequencing. Phylogenetic Investigation of Communities by Reconstruction of Unobserved States (PICRUSt) was used to predict functional transfer of gut microbiota. Gut barrier dysfunction was evaluated by measuring the levels of diamine oxidase (DAO), D-lactic acid (DA), and endotoxin (ET). The serum high-sensitivity C-reactive protein (hs-CRP) level was used to indicate systemic inflammation.

**Results:**

Compared with normal controls, patients with cognitive impairments (AD and MCI) had lower abundance of *Dorea* and higher levels of DAO, DA, and ET. Kyoto Encyclopedia of Genes and Genomes (KEGG) results showed that the pathways related to glycan biosynthesis and metabolism increased in MCI patients, while the ones related to membrane transport decreased. The abundance of *Bacteroides* and *Faecalibacterium* was negatively correlated with the content of ET, and positively correlated with the scores of MMSE and MoCA. The hs-CRP levels were similar among the three groups. A significant negative correlation was observed between the severity of gut barrier dysfunction and cognitive function.

**Conclusion:**

Cognitive impairments might be associated with gut microbial dysbiosis and intestinal barrier dysfunction.

## 1. Introduction

Alzheimer’s disease (AD) is the most common type of dementia, which often presents with progressive cognitive impairment, memory decline, personality changes, and mental abnormalities. The 2011 National Institute on Aging-Alzheimer’s Association (NIA-AA) diagnostic criteria for AD emphasized the continuity of the disease process, with pathophysiological changes beginning 15–20 years before symptoms appear, and divided AD into three stages: preclinical, mild cognitive impairment, and dementia (McKhann et al., 2011). According to the World Alzheimer’s Report, about 46.8 million people currently suffer from dementia worldwide, and the number is expected to exceed 130 million by 2050 (Dixit et al., 2021). At present, drug therapy is still the main treatment method for AD. But the effect is not ideal, and the treatment is often accompanied by multiple adverse reactions. It is urgent to explore new methods to prevent and treat AD.

The gut-brain axis is drawing more attention with a number of researches showing a link between gut microbiome and cognitive dysfunction. Cattaneo found that increases in *Escherichia coli* and *Shigella*, and decrease in *Eubacterium rectale* were possibly associated with inflammation and brain amyloid accumulation in AD (Cattaneo et al., 2017). Based on animal experiments, Chen demonstrated that gut microbiota-targeted photobiomodulation therapy improved senile dementia (Chen et al., 2021). In general, the gut microbiota forms a symbiotic relationship with the intestinal mucosa and the host. The maintenance of gut homeostasis requires normal intestinal barrier function, and once barrier dysfunction occurs, the gut microbiota and their metabolites are prone to translocation, forming “gut leakage” (Pellegrini et al., 2018). Similarly, the dysbiosis of gut microbiome might damage intestinal mucosa, leading to increased permeability and exacerbated microbial imbalances. Therefore, it is reasonable to speculate that gut microbiome and barrier function are related to cognitive defects. This study aimed to investigate these potential correlations between gut microbiota, intestinal barrier function, and cognitive performance, to provide new insights into the prevention and treatment of AD.

## 2. Materials and methods

### 2.1. Study design and subjects

This study followed the Declaration of Helsinki and was approved by the Ethics Committee of PLA Rocket Force Characteristic Medical Center (KY2021035). This research was registered at Chinese Clinical Trial Registry (ChiCTR2100051291).^1^ All participants or their caregivers were informed of the purpose and procedure of this study, and provided their written informed consents.

A total of 118 subjects aged 55–85 years old were recruited in the PLA Rocket Force Characteristic Medical Center from October 2021 to April 2022. The subjects were divided into Alzheimer’s disease (AD), mild cognitive impairment (MCI), and normal control (NC) groups according to the diagnostic criteria of the 2011 National Institute of Aging and Alzheimer’s Association (NIA-AA) (McKhann et al., 2011).

AD patients should meet all the following criteria: Hachinski Ischemic Scale (HIS) score ≤ 4, Clinical Dementia Rating (CDR) score ≥ 1, and Mini Mental State Examination (MMSE) score for education level of junior school or above ≤24, primary school ≤21, illiteracy ≤16. MCI patients were diagnosed based on a memory complaint verified by an informant, normal activities of daily living, MMSE score ≥ 24, and a CDR score of 0.5.

All patients with AD took acetylcholinesterase inhibitors (Donepezil) and/or memantine after diagnosis, whereas those with MCI did not.

The normal control cases were recruited during the same period and were the AD or MCI patients’ family members, who lived together and had similar diet habits with the patients. They all had a MMSE score ≥ 24, a CDR score of 0, and no memory complaints.

The exclusion criteria for all potentially eligible cases in this study were: (1) cases with a history of traumatic brain injury, severe cerebrovascular diseases, or encephalopathy; (2) cases diagnosed with dementia due to the causes other than AD, such as vascular dementia, frontotemporal dementia, and so on; (3) cases with other neurodegenerative diseases, such as Parkinson’s disease or Huntington’s disease; (4) cases with other conditions that may cause cognitive decline, such as hypothyroidism or alcoholism; (5) cases with a history of mental disorders, such as schizophrenia; (6) cases with severe anxiety (Chinese version of Hamilton Anxiety Scale (HAMA) score ≥ 14) or depression (Hamilton Depression Scale (HAMD) score ≥ 20; Sankhe et al., 2017); (7) cases accompanied by severe heart, lung, liver, kidney, blood, or endocrine diseases; (8) cases taking drugs that affect gut microbiome within 3 months, such as antibiotics, probiotics, prebiotics, etc.

### 2.2. Data collection

Through face-to-face interviews and questionnaires, the demographic information and clinical data were collected, including gender, age, years of education, body mass index (BMI), defecation, and disease states and treatment history, etc. According to Bristol stool classification (Blake et al., 2016), patients were assessed to have constipation (type I and type II), be normal (type III and type IV), or have diarrhea (type V to type VII). The nutritional status of the case was evaluated by Mini Nutritional Assessment Short Form (MNA-SF) scale (Liu et al., 2022).

### 2.3. Cognitive function assessment

Cognitive function was mainly assessed by scales including MMSE, MoCA, and CDR, and cognition-related functional ability was evaluated using ADL (Porsteinsson et al., 2021). MMSE scale covers 5 aspects, which are orientation, memory, attention and calculation, recollection, and language ability. As a rapid screening tool for cognitive impairment, the Chinese version of MoCA scale has been broadly used in China and includes 8 cognitive fields, which are attention and concentration, executive function, memory, language, visual structure skills, abstract thinking, calculation, and orientation. CDR scale grades the severity of cognitive impairment by asking the subjects or their caregivers questions about memory, orientation, judgment and problem solving, community affairs, family and hobbies, and personal care. The modified version of ADL scale consists of 20 questions related to daily activities, and subjects are graded on whether and to what extent they need help. The higher the subjects’ score is, the worse the self-care ability is, and the case is more dependent on the care of nursing staff.

### 2.4. Blood-based indicators

All subjects fasted for at least 10 hours overnight before venous blood collection. The collected blood samples were sent to the laboratory department of the hospital (PLA Rocket Force Characteristic Medical Center, Beijing, China) for multiple tests, such as (1) the inflammation indicator high-sensitivity C-reactive protein (hs-CRP), (2) biochemical indicators including total protein (TP), albumin (ALB), total cholesterol (TC), triglyceride (TG), high density lipoprotein (HDL), low density lipoprotein (LDL), fasting blood glucose (GLU), homocysteine (Hcy), alanine transaminase (ALT), total bilirubin (TBIL), direct bilirubin (DBIL), urea (UREA), creatinine (CR), and uric acid (UA), and (3) thyroid hormones including triiodothyronine (TT3), thyroxine (TT4), free triiodothyronine (FT3), free thyroxine (FT4), and thyroid stimulating hormone (TSH). Furthermore, the serum contents of DAO, DA, and ET were measured using a commercial DAO/DA/ET Kit (Beijing Zhongsheng Jinyu Diagnostic Technology Co., Ltd.) according to the instructions (Xie et al., 2019).

### 2.5. 16S rRNA gene sequencing for gut microbiota

All subjects were trained to follow the same procedure to collect stool samples (approximately 10 g),which were stored in −80°C refrigerator before test. The Novogene Co., Ltd. performed the 16S rRNA high-throughput sequencing and bioinformation analysis. Briefly, the fecal genome DNA extraction kits (Tiangen, DP328) were used to extract the DNA from stool samples. The purity and concentration of DNA fragments were determined by spectrophotometer and 1% agarose gel electrophoresis (Ling et al., 2021). The extracted genomic DNA was used as a template, and the V4-V5 region of the bacterial 16S rRNA gene was amplified by PCR with the forward primer 515FB (5′-GTGYCAGCMGCCGCGGTAA-3′) and the reverse primer 926R (5′-CCGYCAATTYMTTTRAGTTT-3′). The sequencing was completed at the Illumina NovaSeq platform, and tags with 97% sequence similarity level were clustered by QIIME 2.0 software to obtain operational taxonomic units (OTUs) against Silva v132 database (Settanni et al., 2021). The raw data were stored in the National Center for Biotechnology Information (NCBI) database with accession number PRJNA946900.

The microbial bioinformatic analysis was conducted by QIIME 2.0 software and R language. The bacterial diversity included α-diversity and β-diversity (Qian et al., 2018). α-diversity helps to measure the number of microbial species in a single sample and the proportion of each species. Four common indexes were used to evaluate α-diversity: Chao1 only reflected the richness of species. Shannon index and Inverse Simpson index represented the richness and evenness of species. Goods Coverage estimated the sequencing depth of the sample. β-diversity is used to measure the similarity of microbial composition among different samples. That is, β-diversity focused on the differences in microbial composition. We selected principal component analysis (PCA) and Bray Curtis for β-diversity analysis.

Based on KEGG functional pathway, the predicted functional composition of gut microbiome in each sample was concluded by Phylogenetic Investigation of Communities by Reconstruction of Unobserved States (PICRUSt). Then the statistical analyses were performed by STAMP, and the differences in orthologs among three groups were compared.

### 2.6. Statistical analysis

Statistical analysis was performed using GraphPad Prism 8.3 (GraphPad Software, Inc.) and R language. The measurement data conforming to normal distribution were expressed as means with standard deviations (means ± SDs) and analyzed using independent sample t test or one-way ANOVA with *post hoc* Tukey pairwise comparison. Otherwise, they were expressed as medians (interquartiles) and compared using Mann–Whitney U test for two groups and Kruskal-Wallis test with *post hoc* Tukey pairwise comparison for more than two groups. The categorical or incidental data were expressed as number and percent and compared using Chi-squared test. Pearson correlation or Spearman rank correlation analysis was performed for investigating the correlations between variables. A *p-*value < 0.05 was considered statistically significant.

## 3. Results

### 3.1. Demographics and clinical data

A total of 118 subjects were enrolled in this study, including 45 AD, 38 MCI, and 35 NC cases. There were 71 females (60.2%) and 47 males (39.8%). The average ages of AD, MCI and NC groups were 71.67, 69.08, and 68.51 years, respectively. There were no significant differences among the three groups in terms of gender (*p* = 0.654), age (*p* = 0.161), duration of education (*p* = 0.667), or BMI (*p* = 0.183). According to Bristol stool classification, stool types differed among the three groups. AD patients had more constipation type (AD vs. MCI vs. NC: 14 vs. 3 vs. 2, *p* < 0.01). The nutrition assessed by the MNA-SF scale showed AD patients had lower scores than the other two groups (mean, AD vs. MCI vs. NC: 11.80 vs. 13.53 vs. 13.71, *p* < 0.01). The difference in hs-CRP level among the three groups was not statistically significant, but it showed an increase from normal controls to patients with AD (mean, NC vs. MCI vs. AD: 0.84 vs. 0.86 vs. 1.10, *p* = 0.146). There were no differences in the prevalence of morbids, anxiety, depression, blood routines, biochemical tests, or thyroid hormone levels among the three groups (*p* > 0.05). All the above results were shown in Table 1.

**Table 1:** Demographics and clinical data.

Characteristics	AD (*n* = 45)	MCI (*n* = 38)	NC (*n* = 35)	*p*-value
Female (n)	29	23	19	0.654
Age (years)	71.67 ± 8.33	69.08 ± 7.16	68.51 ± 8.19	0.161
Education (years)	11.13 ± 3.54	11.08 ± 3.14	11.77 ± 4.29	0.667
BMI (kg/m^2^)	24.97 ± 4.04	25.19 ± 2.60	26.40 ± 3.90	0.183
Constipation (n)	14^**,##^	3	2	<0.01
MNA-SF (scores)	11.80 ± 1.44^**,##^	13.53 ± 0.83	13.71 ± 0.67	<0.01
Hyperlipidemia (n)	28	26	23	0.838
Diabetes (n)	13	8	6	0.439
Hypertension (n)	23	19	17	0.975
HAMA (scores)	7.09 ± 3.50	7.32 ± 3.26	6.60 ± 3.47	0.660
HAMD (scores)	9.47 ± 3.88	9.74 ± 3.50	8.06 ± 3.49	0.112
hs-CRP (mg/L)	1.10 ± 0.78	0.86 ± 0.58	0.84 ± 0.54	0.146
TP (g/L)	71.29 ± 5.98	71.50 ± 4.54	71.83 ± 3.59	0.888
ALB (g/L)	42.69 ± 4.42	43.85 ± 1.97	44.47 ± 2.79	0.053
TC (mmol/L)	4.35 ± 1.10	4.35 ± 1.28	4.15 ± 1.35	0.729
TG (mmol/L)	1.44 ± 0.88	1.45 ± 0.93	1.39 ± 0.89	0.954
HDL (mmol/L)	1.32 ± 0.49	1.30 ± 0.35	1.20 ± 0.36	0.383
LDL (mmol/L)	2.56 ± 0.89	2.57 ± 1.03	2.47 ± 1.08	0.899
GLU (mmol/L)	5.92 ± 1.79	5.84 ± 1.29	5.43 ± 0.79	0.255
Hcy (μmol/L)	11.21 ± 5.46	10.26 ± 4.21	9.98 ± 4.20	0.467
ALT (U/L)	15.31 ± 7.56	16.92 ± 8.36	19.43 ± 8.19	0.078
TBIL (μmol/L)	11.03 ± 4.41	12.04 ± 5.37	13.32 ± 8.29	0.252
DBIL (μmol/L)	3.65 ± 1.58	3.88 ± 1.88	4.24 ± 2.13	0.365
UREA (mmol/L)	5.41 ± 1.72	5.13 ± 1.61	5.27 ± 1.62	0.742
CR (μmol/L)	68.41 ± 16.84	69.98 ± 21.04	65.48 ± 20.60	0.606
UA (μmol/L)	330.46 ± 116.67	287.74 ± 86.93	297.99 ± 75.79	0.112
TT3 (ng/mL)	1.11 ± 0.25	1.18 ± 0.29	1.25 ± 0.28	0.080
TT4 (μg/dL)	6.66 ± 1.17	6.89 ± 1.12	6.97 ± 1.39	0.507
FT3 (pg/mL)	2.73 ± 0.44	2.92 ± 0.59	2.83 ± 0.43	0.233
FT4 (ng/dL)	1.17 ± 0.18	1.20 ± 0.17	1.23 ± 0.21	0.447
TSH (μIU/mL)	2.32 ± 1.14	2.19 ± 1.16	2.36 ± 0.88	0.769

Data were expressed as mean with standard deviation (mean ± SD) or number (n). ^*^p < 0.05 and ^**^p < 0.01 compared with NC. ^#^*p* < 0.05 and ^##^*p* < 0.01 compared with MCI group. AD, Alzheimer’s disease; MCI, mild cognitive impairment; NC, normal control.

### 3.2. Cognitive function

The scores of MMSE, MoCA, and ADL were significantly different among the three groups (*p* < 0.01). The mean scores of MMSE were 16.80 in AD group, 27.42 in MCI group, and 29.06 in NC group, respectively. In the score of MoCA, AD group and MCI group had significantly lower scores than the NC group (*p* < 0.01). AD patients had the highest score of ADL (*p* < 0.01). The above results were detailed in Table 2.

**Table 2:** Cognitive function.

Scale	AD (*n* = 45)	MCI (*n* = 38)	NC (*n* = 35)	*P*-value
MMSE score	16.80 ± 6.98^**,##^	27.42 ± 1.22^**^	29.06 ± 0.91	<0.01
MoCA score	13.27 ± 6.07^**,##^	21.58 ± 2.84^**^	27.03 ± 1.32	<0.01
ADL score	39.31 ± 13.71^**,##^	23.21 ± 1.32^**^	20.00 ± 0.00	<0.01

Data were expressed as mean with standard deviation (mean ± SD). ^*^p < 0.05 and ^**^p < 0.01 compared with NC. ^#^*p* < 0.05 and ^##^*p* < 0.01 compared with MCI group. AD, Alzheimer’s disease; MCI, mild cognitive impairment; NC, normal control.

### 3.3. Gut microbiome dysbiosis

**Figure 1:**
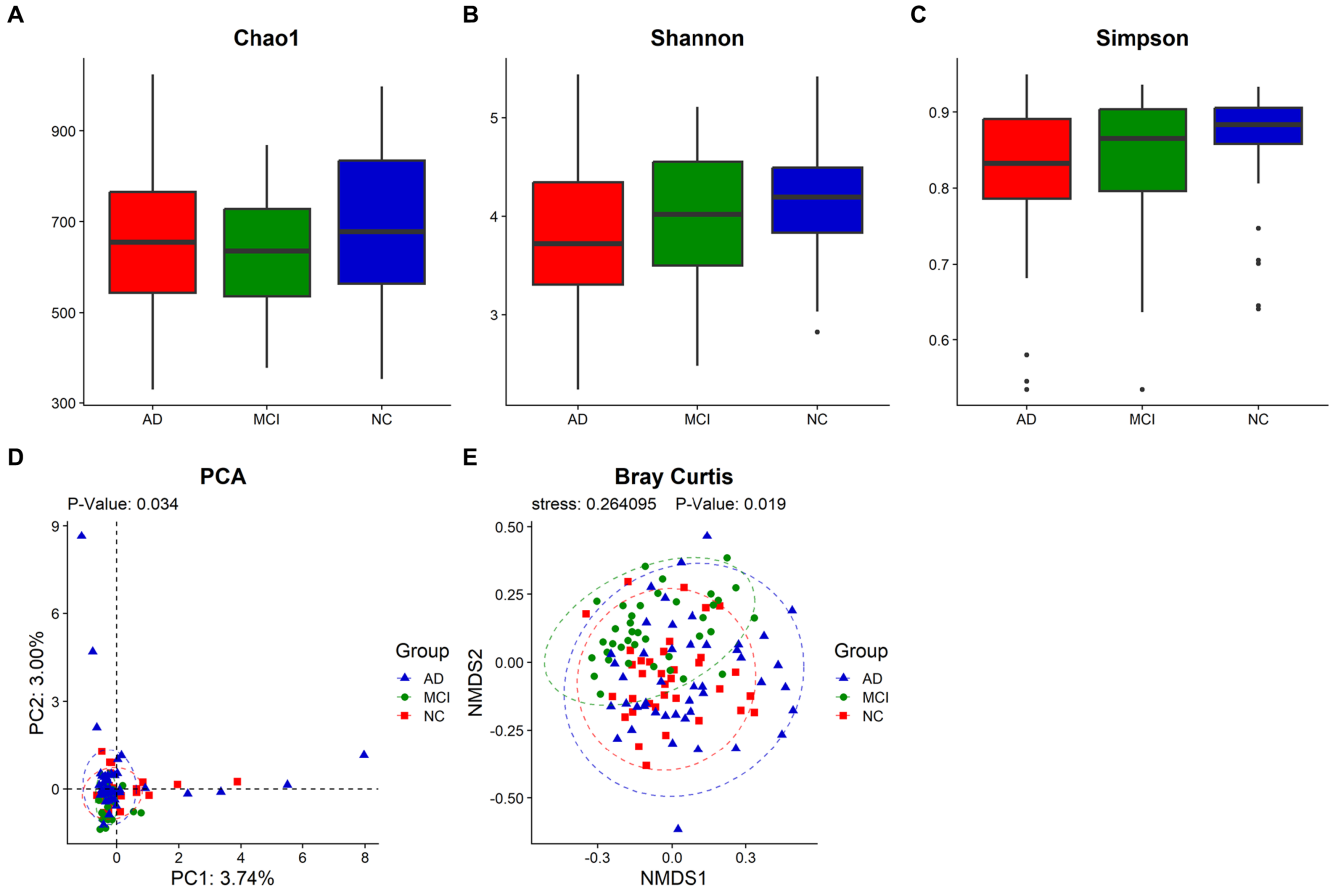
The diversity analysis of the gut microbiome. The α-diversity was represented by Chao1 **(A)**, Shannon index **(B)** and Inverse Simpson index **(C)**, and the β-diversity was described by PCA **(D)** and Bray Curtis **(E)**. Each box plot represented the median, interquartile range, minimum, and maximum values. *p* < 0.05 was considered statistically significant.

The Goods Coverage revealed that each individual had most of gut microbiome members (Supplementary Figure S1). The Venn diagram showed that 2,204 OTUs of the total 4,887 were shared among all samples, and that AD, MCI, and NC groups possessed 632, 332, and 201 specific OTUs, respectively, (Supplementary Figure S2). Although the α-diversity of gut microbiome did not show significant differences among the three groups (Chao1: *p* = 0.259; Shannon index: *p* = 0.138; Inverse Simpson index: *p* = 0.123), Shannon index and Inverse Simpson index of patients with cognitive impairment were lower than those of normal controls (median, Shannon index, AD vs. MCI vs. NC: 3.72 vs. 4.02 vs. 4.20; Inverse Simpson index, AD vs. MCI vs. NC: 0.83 vs. 0.87 vs. 0.88; Figures 1A–C). Principal component analysis (PCA) and Bray Curtis analysis implied the differences in β-diversity (PCA, *p* = 0.034; Bray Curtis, *p* = 0.019) among the three groups (Figures 1D,E).

**Figure 2:**
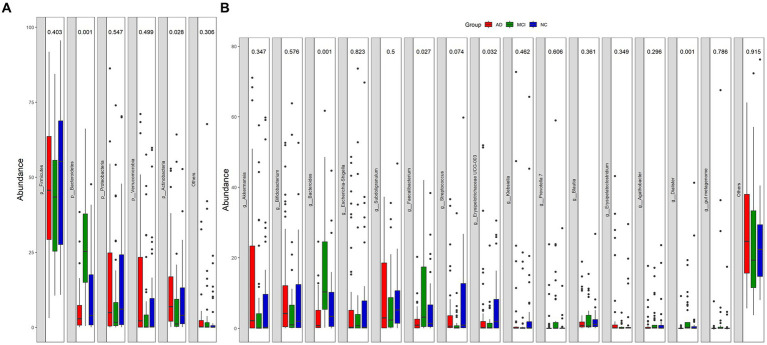
Relative abundance of gut microbial taxa at phylum-level **(A)** and genus-level **(B)**.

**Figure 3:**
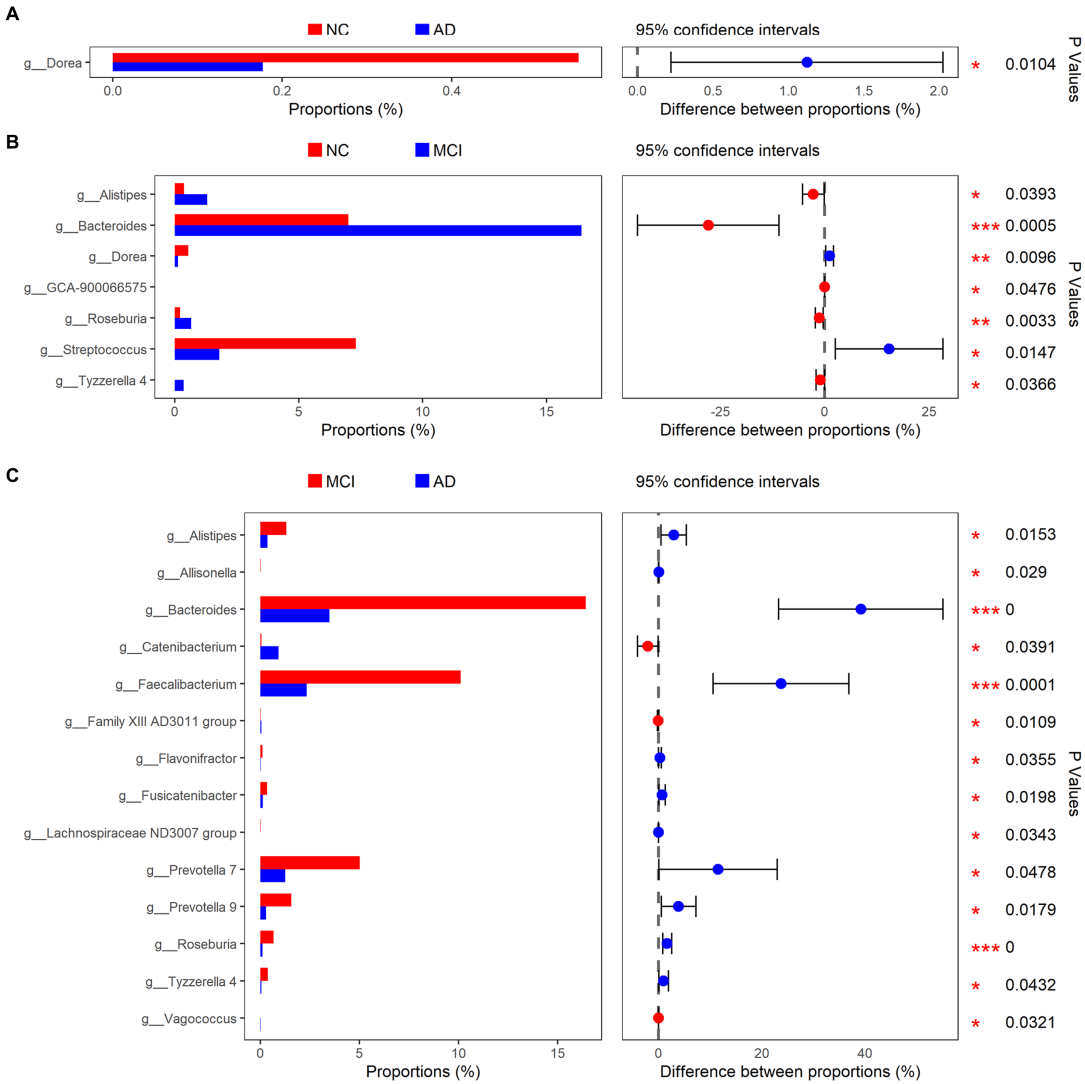
Bacterial genus with different relative abundance in NC/AD groups **(A)**, NC/MCI groups **(B)**, and MCI/AD groups **(C)**.

At the phylum level, Firmicutes, Bacteroidetes, Proteobacteria, Verrucomicrobia, and Actinobacteria were the main bacteria types for all samples (Figure 2A). There were significant differences in Bacteroidetes and Actinobacteria among the three groups (Bacteroidetes: *p* < 0.01; Actinobacteria: *p* = 0.028; Figure 2A). At the genus level, *Akkermansia*, *Bifidobacterium*, *Bacteroides*, *Escherichia-Shigella*, *Subdoligranulum*, *Faecalibacterium*, *Streptococcus*, and *Erysipelotrichaceae* UCG-003 were the dominant genera (Figure 2B). The abundance of *Dorea* was greatly lower in patients with cognitive impairment than that in normal controls (mean, AD vs. MCI vs. NC: 0.17% vs. 0.13% vs. 0.54%, *p* < 0.01; Figures 3A,B). Unexpectedly, MCI group had more *Alistipes*, *Bacteroides*, *Roseburia*, and *Tyzzerella* 4 than the other two groups (Figures 3B,C). In addition to the bacteria mentioned above, MCI group also had higher abundance of *Allisonella* (*p* = 0.029), *Faecalibacterium* (*p* < 0.01), *Flavonifractor* (*p* = 0.036), *Fusicatenibacter* (*p* = 0.020), *Lachnospiraceae ND3007 group* (*p* = 0.034), *Prevotella* 7 (*p* = 0.048), and *Prevotella* 9 (*p* = 0.018) compared with AD group (Figure 3C). In contrast, *Catenibacterium* (*p* = 0.039), *Family XIII AD3011 group* (*p* = 0.011), and *Vagococcus* (*p* = 0.032) were more abundant in AD (Figure 3C). Besides, normal controls also had more *Streptococcus* than patients with MCI (*p* = 0.015; Figure 3B).

### 3.4. Predicted functional analysis of microbiome

To further prove the connection between intestinal microbiota and brain in cognitive impairment, KEGG functional orthologs were predicted with PICRUSt. There were a wide range of potential communication pathways between gut microbiome and patients with cognitive impairment, including cellular processes, environmental information processing, genetic information processing, human diseases, metabolism, and organismal systems, as shown in Table 3. Compared with NC group, there were 15 significant changes of level 2 KEGG pathways in patients with MCI, including the increases of cell growth and death, transport and catabolism, folding, sorting and degradation, etc. Similarly, compared with NC, 13 functional orthologs were altered in AD patients, among which the enriched orthologs in AD patients were cellular community (eukaryotes), information processing in viruses, transcription, drug resistance (antineoplastic), and infectious disease (viral). In addition, AD patients had 17 altered modules compared with MCI patients, including enriched ones related to cellular community (prokaryotes), membrane transport, and infectious disease (parasitic), etc.

**Table 3:** Predicted KEGG functional pathways differences at level 2.

KO functional categories		AD mean% (SD%)	MCI mean% (SD%)	NC mean% (SD%)	*P*-value		
Level 1	Level 2				AD vs. MCI	AD vs. NC	MCI vs. NC
Cellular processes	Cell growth and death	1.14 (0.20)	1.29 (0.18)	1.19 (0.17)	<0.01	0.021	0.016
Cellular processes	Cellular community—eukaryotes	0.00 (0.00)	0.00 (0.00)	0.00 (0.00)	–	<0.01	–
Cellular processes	Cellular community—prokaryotes	3.12 (0.67)	2.67 (0.65)	3.02 (0.60)	<0.01	–	<0.01
Cellular processes	Transport and catabolism	0.23 (0.10)	0.29 (0.13)	0.24 (0.11)	<0.01	–	0.020
Environmental information processing	Membrane transport	9.14 (1.70)	7.94 (1.47)	8.94 (1.60)	<0.01	–	<0.01
Environmental information processing	Signaling molecules and interaction	0.00 (0.00)	0.00 (0.00)	0.00 (0.00)	–	0.010	–
Genetic information processing	Folding, sorting and degradation	2.44 (0.23)	2.58 (0.21)	2.46 (0.21)	<0.01	–	<0.01
Genetic information processing	Information processing in viruses	0.01 (0.01)	0.01 (0.01)	0.01 (0.01)	–	0.017	–
Genetic information processing	Transcription	0.51 (0.14)	0.50 (0.14)	0.48 (0.09)	–	0.012	–
Human diseases	Cardiovascular disease	0.17 (0.02)	0.17 (0.02)	0.17 (0.02)	0.042	–	–
Human diseases	Drug resistance, antimicrobial	1.42 (0.27)	1.48 (0.23)	1.48 (0.23)	–	0.035	–
Human diseases	Drug resistance, antineoplastic	0.11 (0.02)	0.11 (0.02)	0.11 (0.02)	–	0.018	–
Human diseases	Infectious disease, parasitic	0.05 (0.03)	0.04 (0.02)	0.05 (0.02)	0.039	–	–
Human diseases	Infectious disease, viral	0.04 (0.12)	0.03 (0.07)	0.01 (0.00)	–	<0.01	–
Human diseases	Neurodegenerative disease	0.13 (0.04)	0.15 (0.03)	0.12 (0.02)	<0.01	–	<0.01
Metabolism	Biosynthesis of other secondary metabolites	0.79 (0.13)	0.87 (0.14)	0.82 (0.11)	<0.01	–	0.044
Metabolism	Global and overview maps	32.42 (1.00)	33.14 (1.20)	32.58 (0.99)	<0.01	–	<0.01
Metabolism	Glycan biosynthesis and metabolism	2.53 (0.32)	2.89 (0.43)	2.59 (0.40)	<0.01	–	<0.01
Metabolism	Lipid metabolism	1.68 (0.09)	1.73 (0.12)	1.71 (0.09)	0.020	<0.01	–
Metabolism	Metabolism of cofactors and vitamins	3.38 (0.26)	3.62 (0.29)	3.46 (0.21)	<0.01	<0.01	<0.01
Metabolism	Metabolism of terpenoids and polyketides	0.69 (0.06)	0.71 (0.07)	0.69 (0.06)	–	–	0.031
Organismal systems	Development and regeneration	0.01 (0.00)	0.01 (0.00)	0.01 (0.00)	0.037	0.021	-
Organismal systems	Digestive system	0.12 (0.04)	0.15 (0.04)	0.12 (0.04)	<0.01	–	<0.01
Organismal systems	Endocrine system	0.26 (0.05)	0.28 (0.05)	0.26 (0.04)	–	–	0.048
Organismal systems	Immune system	0.09 (0.02)	0.10 (0.02)	0.09 (0.02)	<0.01	<0.01	<0.01
Organismal systems	Nervous system	0.06 (0.01)	0.07 (0.01)	0.06 (0.01)	<0.01	<0.01	<0.01

Data were expressed as mean% (SD%). AD, Alzheimer’s disease; MCI, mild cognitive impairment; NC, normal control; KO, KEGG Ortholog.

### 3.5. Gut barrier dysfunction

The levels of diamine oxidase (DAO), D-lactic acid (DA), and bacterial endotoxin (ET) represented the extent of gut barrier dysfunction. Compared with normal controls, patients with cognitive impairment had higher levels of DAO (mean, AD vs. MCI vs. NC: 11.66 vs. 10.81 vs. 10.47, *p* = 0.034), DA (mean, AD vs. MCI vs. NC: 13.30 vs. 12.43 vs. 11.14, *p* < 0.01), and ET (mean, AD vs. MCI vs. NC: 20.99 vs. 18.21 vs. 17.01, *p* < 0.01). The above results were revealed in Table 4.

**Table 4:** Gut barrier dysfunction.

Biomarkers	AD (*n* = 45)	MCI (*n* = 38)	NC (*n* = 35)	*P*-value
Diamine oxidase (U/L)	11.66 ± 1.91^*^	10.81 ± 2.24	10.47 ± 1.76	0.034
D-lactic acid (mg/L)	13.30 ± 2.27^**^	12.43 ± 2.01^*^	11.14 ± 1.78	<0.01
Endotoxin (U/L)	20.99 ± 2.34^**,##^	18.21 ± 2.23^*^	17.01 ± 1.37	<0.01

Data were expressed as mean with standard deviation (mean ± SD). ^*^p < 0.05 and ^**^p < 0.01 compared with NC. ^#^*p* < 0.05 and ^##^*p* < 0.01 compared with MCI group. AD, Alzheimer’s disease; MCI, mild cognitive impairment; NC, normal control.

### 3.6. Association between intestinal microbiota and gut barrier dysfunction/inflammation/cognitive function

**Figure 4:**
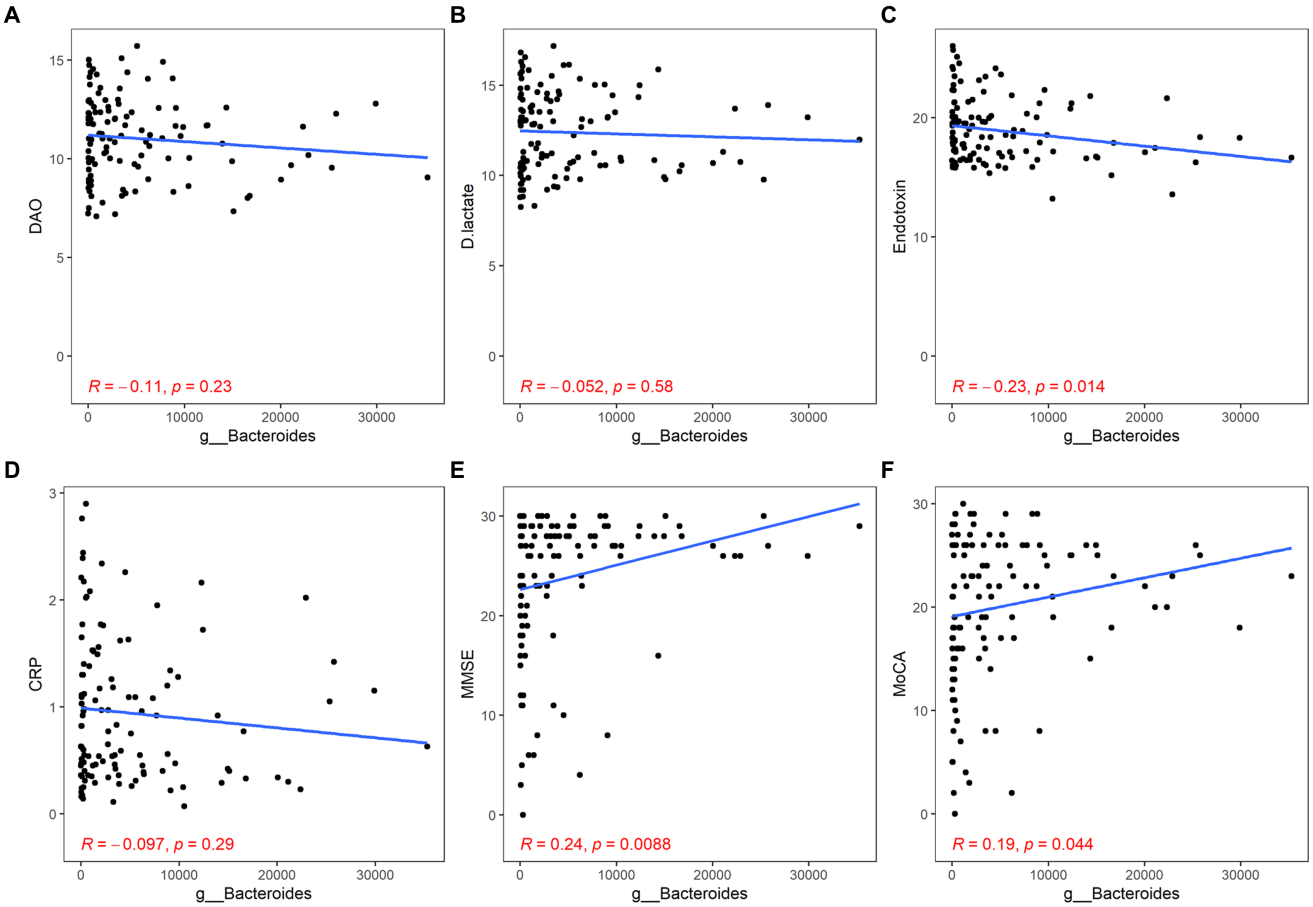
Correlation between *Bacteroides* and gut barrier dysfunction/inflammation/cognitive function. The gut barrier dysfunction was evaluated by DAO **(A)**, DA **(B)** and ET **(C)**. The content of hs-CRP was used to estimate systemic inflammation **(D)**. The cognitive function was described by the scores of MMSE **(E)** and MoCA **(F)**. *p* < 0.05 was considered statistically significant.

**Figure 5:**
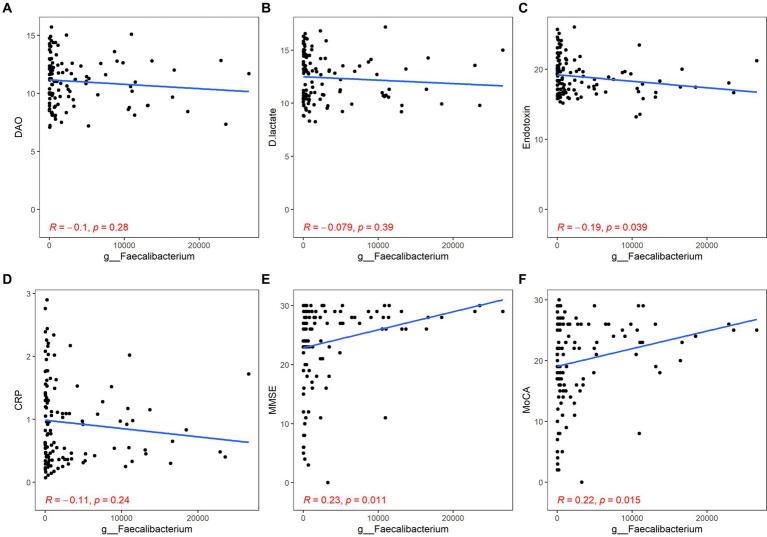
Correlation between *Faecalibacterium* and gut barrier dysfunction/inflammation/cognitive function. The gut barrier dysfunction was evaluated by DAO **(A)**, DA **(B),** and ET **(C)**. The content of hs-CRP was used to estimate systemic inflammation **(D)**. The cognitive function was described by the scores of MMSE **(E)** and MoCA **(F)**. *p* < 0.05 was considered statistically significant.

Both *Bacteroides* and *Faecalibacterium* abundance were negatively correlated with the content of ET and positively correlated with the scores of MMSE and MoCA, but their correlation with inflammation was not statistically significant (*Bacteroides*: ET, *r* = −0.23, *p* = 0.014; MMSE, *r* = 0.24, *p* < 0.01; MoCA, *r* = 0.19, *p* = 0.044; *Faecalibacterium*: ET, *r* = −0.19, *p* = 0.039; MMSE, *r* = 0.23, *p* = 0.011; MoCA, *r* = 0.22, *p* = 0.015; Figures 4, 5).

### 3.7. Association between gut barrier dysfunction and cognitive function

**Figure 6:**
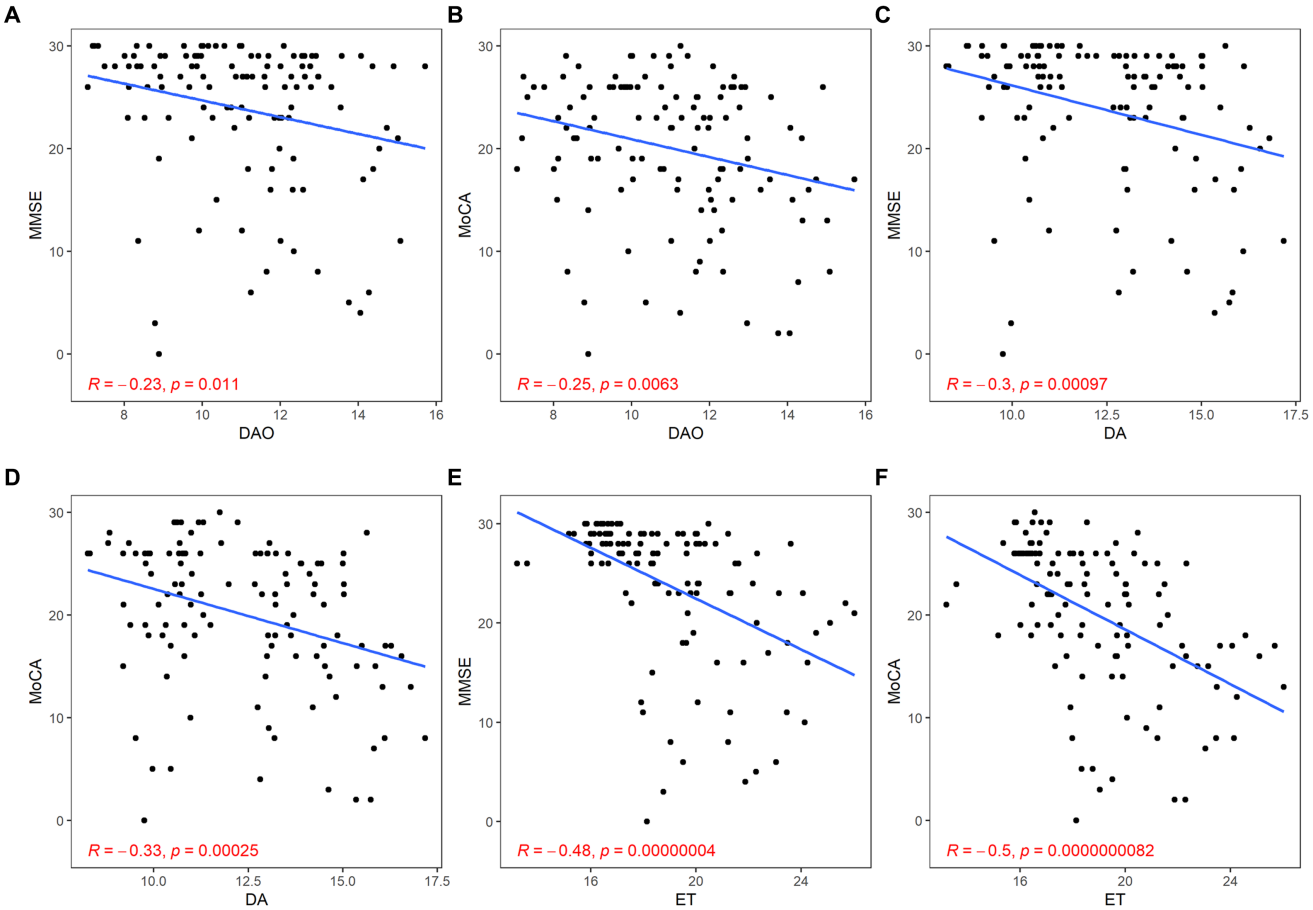
Correlation between gut barrier dysfunction and cognitive function. The gut barrier dysfunction was evaluated by DAO **(A,B)**, DA **(C,D),** and ET **(E,F)**. The cognitive function was described by the scores of MMSE **(A,C,E)** and MoCA **(B,D,F)**. *p* < 0.05 was considered statistically significant.

There was a significant negative correlation between barrier impairment and cognitive scores (DAO: MMSE, *r* = −0.23, *p* = 0.011; MoCA, *r* = −0.25, *p* < 0.01; DA: MMSE, *r* = −0.30, *p* < 0.01; MoCA, *r* = −0.33, *p* < 0.01; ET: MMSE, *r* = −0.48, *p* < 0.01; MoCA, *r* = −0.50, *p* < 0.01; Figure 6).

## 4. Discussion

In this cross-sectional study, we observed altered microbiome and abnormal gut barrier function in subjects with cognitive impairment. Compared with normal controls, patients with AD had higher β-diversity in microbial composition. The abundance of *Dorea* in MCI and AD groups was greatly lower than that in NC group. Meanwhile, patients with cognitive impairment had apparent gut barrier dysfunction. Through further analysis, the abundance of *Bacteroides* and *Faecalibacterium* was negatively correlated with the content of endotoxin and positively correlated with the scores of MMSE and MoCA. The levels of DAO, DA, and ET were negatively correlated with the scores of MMSE and MoCA. These results indicated that gut microbiota dysbiosis and barrier dysfunction maybe associated with the development of AD.

The difference in β-diversity meant that each group had its own unique microbial composition, which proved that our study based on microbiota was feasible. However, there was no significant difference in α-diversity among the three groups, which was consistent with Guo’s study (Guo et al., 2021). Findings about gut microbial α-diversity in dementia have been contradictory. Vogt found a decline in α-diversity among AD patients (Vogt et al., 2017), but a Japanese study showed an increase (Saji et al., 2019). That might be due to different geographical locations and microbial sequencing techniques (Gao et al., 2021). The exact reasons for changes in the gut bacterial diversity of AD patients are still unclear. Some scholars have speculated that gut microbes in dementia are not as stable as those in healthy people, and are more susceptible to external disruption, resulting in the imbalance of microbiota and increased susceptibility to amyloid plaques accumulation, which ultimately promotes the development of AD (Shabbir et al., 2021).

When comparing the microbial composition, *Dorea* was the only difference between AD and NC groups, which might be related to the low degree of dementia in the AD patients we included and the long-term use of therapeutic drugs, such as Donepezil and memantine (Li et al., 2019). We observed that the abundance of *Dorea* in patients with cognitive impairment was lower than that in normal controls, which was contrary to Liu’s findings (Liu et al., 2019). This might be caused by the difference in diet between the southern and northern parts of China. Liu’s subjects were from the south, who had a light diet and often took vegetables and fruits, while our participants came from the north, who usually ate high-calorie food, such as fried food and dumplings. It has been confirmed that long-term consumption of fried food can reduce the abundance of *Dorea* and alter the distribution of fecal metabolites (Gao et al., 2021).

Functional interpretation of metagenomes suggested that cognitive impairment was associated with various modulations of functional KEGG pathways, mainly involved in membrane transport, neurodegenerative disease, glycan biosynthesis and metabolism, digestive system, immune system, and nervous system. These results supported that gut microbes influence brain through neurological, endocrine, and immune pathways, which were consistent with previous studies (Mohajeri et al., 2018). It was worth noting that compared with normal controls, medicated AD patients showed significant declines in the related homologs of glycan biosynthesis and metabolism, digestive system, immune system, and nervous system, while untreated MCI patients showed increases. These functional results suggested that gut microbiota might play a role in metabolic disorders and immune activation in the patients with cognitive impairment, and that therapeutic drug could also influence these pathways to produce therapeutic effects, providing new insights into the prevention and treatment of cognitive impairment (De-Paula et al., 2018).

Through correlation analysis, we found that *Bacteroides* and *Faecalibacterium* were both negatively correlated with content of endotoxin and positively correlated with scores of cognitive assessment scales, suggesting that these bacteria were positively related to intestinal barrier and cognition. The influence of *Bacteroides* on the brain can be traced back to the infant period. A Canadian study found that an enrichment of *Bacteroides* in late infancy boosted neural development (Tamana et al., 2021). *Bacteroides* and *Faecalibacterium* were likely to affect brain and gut function through their metabolites, short-chain fatty acids (SCFAs). SCFAs are mainly produced by intestinal microbial colysis after intake of dietary fiber, including acetate, propionate and butyrate. Both acetate and propionate can inhibit the release of pro-inflammatory cytokines from neutrophils and macrophages, which may play an anti-inflammatory role (He et al., 2020). Butyrate may enhance gut barrier and blood–brain barrier (BBB) by increasing expression of tight-junction proteins and producing antimicrobial peptides (Silva et al., 2020). Meanwhile, it can regulate expression of brain-derived neurotrophic factor (BDNF) and N-methyl-D-aspartic acid (NMDA) receptors, promote neurogenesis, and participate in the formation of synaptic plasticity (Oroojzadeh et al., 2022). It also facilitates serotonin formation and improves neuronal homeostasis and function. In addition, SCFAs are also essential for proper intestinal function (Ma et al., 2022). Recent studies have shown that the alterations of gut microbiome in AD may damage intestinal epithelial cells via the anti-inflammatory P-glycoprotein pathway, so that gut bacteria and their metabolites could enter the blood and increase the risk of lipopolysaccharide (LPS) deposition in the brain (Haran et al., 2019). A longitudinal study observed that patients with inflammatory bowel disease (IBD) were more than twice likely to develop dementia (Zhang et al., 2021). A clinical trial showed that probiotics could improve cognitive function and metabolic statuses in the patients with AD (Akbari et al., 2016). Another controlled trial demonstrated that *Bifidobacterium* as a probiotic might improve cognition and prevent brain atrophy in MCI (Asaoka et al., 2022). *Faecalibacterium prausnitzii* (*F. prausnitzii*), as one of the important producers of butyrate, has anti-inflammatory effects and maintains the activity of bacterial enzymes, which is seen as a candidate for the next generation of probiotics (Gebrayel et al., 2022). This seems to explain why its abundance is higher in MCI than in AD. In conclusion, gut microbiome dysbiosis will damage gut barrier and blood–brain barrier through multiple pathways, trigger neuroinflammation, and promote apoptosis of neurons and glial cells, especially in the hippocampus and cerebral cortex, which may be the basis for the development of AD (Liu et al., 2020).

In clinical work, gut barrier function is often tested indirectly, such as assessing barrier permeability via measuring the contents of endotoxin and diamine oxidase in blood, conducting bacterial culture to observe whether there is bacterial migration, and determining the PH value of intestinal mucosa (Schoultz and Keita, 2020). We chose to use the DAO/DA/ET kit (enzymatic assay) to evaluate gut barrier function. We found that the contents of diamine oxidase, D-lactate and endotoxin were significantly higher in the patients with cognitive impairment than those in normal controls, which suggested these patients had gut barrier dysfunction. Further correlation analysis confirmed that intestinal barrier damage was negatively correlated with cognitive function. Similarly, Stadlbauer’s study discovered that the patients with dementia had increased levels of DAO and soluble cluster differentiation 14 (sCD14; Stadlbauer et al., 2020). Park also found that the levels of IL-1β and TGF-β in the AD group were significantly higher than those in the MCI and NC groups (Park et al., 2021). However, the level of hs-CRP, an indicator of systemic inflammation in our study, did not differ markedly from the three groups. This might be related to the lower severity of dementia in the patients we included (Leblhuber et al., 2021).

With the increase of age, the various functions of the human body will decline more or less. Aging itself is associated with cognitive decline, intestinal ecological imbalance, increased mucosal permeability, inflammatory stress, and bacterial translocation (Komanduri et al., 2019). A special marker of successful aging may be the ability of the microbiome to maintain or up-regulate anti-inflammatory activity by keeping a balance between pro-inflammatory and anti-inflammatory responses in the body, which is a characteristic of long-lived elderly people (Badal et al., 2020). To exclude the influence of age (aging) and gender, we included normal persons with similar age and gender composition to the patients. In addition, diet may be the most critical factor to the composition of gut microbiome (Gomaa, 2020). In order to minimize the impact of diet, we included patients’ healthy spouses or long-term caregivers in the control group as much as possible, because they lived together and had the same diet daily.

We observed that the subjects with cognitive impairment were more likely to be accompanied by constipation, which was consistent with many studies (Chen et al., 2020; Wang et al., 2022). The patients with dementia have limited movement, slowed bowel movement, and drug abuse, which makes defecation more difficult. Constipation will hinder the excretion of toxins in the body, which might cause harm to human body, especially the brain, thus forming a vicious cycle (Chen et al., 2020).

In this study, the MNA-SF score of the AD group was significantly lower than that of the MCI and the NC groups, indicating that the nutritional status was poor. Adequate nutrition helps to ensure the structure and function of the brain as much as possible and delay cognitive decline (Kawashima, 2021). The elderly, especially those who have been bedridden for a long time, are more likely to suffer from dementia due to their poor nutritional status (Brockdorf and Morley, 2021). We should pay more attention to nutritional care of the elderly, especially those with dementia.

There were some limitations in our study. Firstly, single-center recruitment and small sample size might cause bias. Secondly, although we excluded the patients that took antibiotics and probiotics, there was still the possibility that other unknown drugs might interfere with the gut microbiota. Thirdly, this was a cross-sectional observational study and could not prove a causal relationship between cognitive function and gut microbiota and gut barrier. More large-sample longitudinal studies were needed.

## 5. Conclusion

In conclusion, this study indicated the patients with cognitive impairment had the alterations in gut microbial composition and damage to the intestinal barrier. Regulating the gut microbiome and strengthening barrier function might be a new treatment method for dementia in the future if more researches could solidify the current findings.

## Data availability statement

The original contributions presented in the study are publicly available. This data can be found here: https://www.ncbi.nlm.nih.gov/, PRJNA946900.

## Ethics statement

This study was approved by the Ethics Committee of PLA Rocket Force Characteristic Medical Center (KY2021035). The patients/participants provided their written informed consent to participate in this study.

## Author contributions

YP: writing the initial draft, design of methodology, investigation, and revising the manuscript. YL and HL: designing the methods and revising the manuscript. CJ: funding acquisition, designing the methods, visualization and interpretation of data, and revising the manuscript. LW: design of the study, supervision, and revising the manuscript. All authors contributed to the article and approved the submitted version.

## Conflict of interest

The authors declare that the research was conducted in the absence of any commercial or financial relationships that could be construed as a potential conflict of interest.

## Publisher’s note

All claims expressed in this article are solely those of the authors and do not necessarily represent those of their affiliated organizations, or those of the publisher, the editors and the reviewers. Any product that may be evaluated in this article, or claim that may be made by its manufacturer, is not guaranteed or endorsed by the publisher.

## Footnotes

^1^https://www.chictr.org.cn/
