# Supplementary material for: Gut microbiota and intestinal barrier function in subjects with cognitive impairments: a cross-sectional study
Source: Front Aging Neurosci. 2023 Jun 7;15:1174599. doi: 10.3389/fnagi.2023.1174599 (PMC10282132; doi:10.3389/fnagi.2023.1174599)
Supplement: Supplementary file 1 [file Data_Sheet_1.docx]

Supplementary Material

# Supplementary Data

**Table 1 Demographic and clinical data**

| **Characteristics** | **AD**  **(n=45)** | **MCI**  **(n=38)** | **NC**  **(n=35)** | ***P*-value** |
| --- | --- | --- | --- | --- |
| **Female (n)** | 29 | 23 | 19 | 0.654 |
| **Age (years)** | 71.67±8.33 | 69.08±7.16 | 68.51±8.19 | 0.161 |
| **Education (years)** | 11.13±3.54 | 11.08±3.14 | 11.77±4.29 | 0.667 |
| **BMI (kg/m^2^)** | 24.97±4.04 | 25.19±2.60 | 26.40±3.90 | 0.183 |
| **Constipation (n)** | 14**## | 3 | 2 | < 0.01 |
| **MNA-SF (scores)** | 11.80±1.44**## | 13.53±0.83 | 13.71±0.67 | < 0.01 |
| **Hyperlipidemia (n)** | 28 | 26 | 23 | 0.838 |
| **Diabetes (n)** | 13 | 8 | 6 | 0.439 |
| **Hypertension (n)** | 23 | 19 | 17 | 0.975 |
| **HAMA (scores)** | 7.09±3.50 | 7.32±3.26 | 6.60±3.47 | 0.660 |
| **HAMD (scores)** | 9.47±3.88 | 9.74±3.50 | 8.06±3.49 | 0.112 |
| **hs-CRP (mg/L)** | 1.10±0.78 | 0.86±0.58 | 0.84±0.54 | 0.146 |
| **TP (g/L)** | 71.29±5.98 | 71.50±4.54 | 71.83±3.59 | 0.888 |
| **ALB (g/L)** | 42.69±4.43 | 43.85±1.97 | 44.47±2.79 | 0.053 |
| **TC (mmol/L)** | 4.35±1.10 | 4.35±1.28 | 4.15±1.35 | 0.729 |
| **TG (mmol/L)** | 1.44±0.88 | 1.45±0.93 | 1.39±0.89 | 0.954 |
| **HDL (mmol/L)** | 1.32±0.49 | 1.30±0.35 | 1.20±0.36 | 0.383 |
| **LDL (mmol/L)** | 2.56±0.89 | 2.57±1.03 | 2.47±1.08 | 0.899 |
| **GLU (mmol/L)** | 5.92±1.79 | 5.84±1.29 | 5.43±0.79 | 0.255 |
| **Hcy (μmol/L)** | 11.21±5.46 | 10.26±4.21 | 9.98±4.21 | 0.467 |
| **ALT (U/L)** | 15.31±7.56 | 16.92±8.36 | 19.43±8.19 | 0.078 |
| **TBIL (μmol/L)** | 11.03±4.41 | 12.04±5.37 | 13.32±8.29 | 0.252 |
| **DBIL (μmol/L)** | 3.65±1.58 | 3.88±1.88 | 4.24±2.13 | 0.365 |
| **UREA (mmol/L)** | 5.41±1.72 | 5.13±1.61 | 5.27±1.62 | 0.742 |
| **CR (μmol/L)** | 68.41±16.84 | 69.98±21.04 | 65.48±20.60 | 0.606 |
| **UA (μmol/L)** | 330.46±116.67 | 287.74±86.93 | 297.99±75.79 | 0.112 |
| **TT3 (ng/mL)** | 1.11±0.25 | 1.18±0.29 | 1.25±0.28 | 0.080 |
| **TT4 (μg/dL)** | 6.66±1.17 | 6.89±1.12 | 6.97±1.39 | 0.507 |
| **FT3 (pg/mL)** | 2.73±0.44 | 2.92±0.59 | 2.83±0.43 | 0.233 |
| **FT4 (ng/dL)** | 1.17±0.18 | 1.20±0.17 | 1.23±0.21 | 0.447 |
| **TSH (μIU/mL)** | 2.32±1.14 | 2.19±1.16 | 2.36±0.88 | 0.769 |

Note: Data expressed as number with case (n) , or means with standard deviation (mean±SD) . *p < 0.05 and **p < 0.01 compared with NC; #p < 0.05 and ##p < 0.01 compared with MCI group. Abbreviations: AD, Alzheimer’s disease; MCI, mild cognitive impairment; NC, normal control.

**Table 2 Cognitive function assessment**

| **Scale** | **AD**  **(n=45)** | **MCI**  **(n=38)** | **NC**  **(n=35)** | ***P*-value** |
| --- | --- | --- | --- | --- |
| **MMSE (scores)** | 16.80±6.98**## | 27.42±1.22** | 29.06±0.91 | < 0.01 |
| **MoCA (scores)** | 13.27±6.07**## | 21.58±2.84** | 27.03±1.32 | < 0.01 |
| **ADL (scores)** | 39.31±13.71**## | 23.21±1.32** | 20.00±0.00 | < 0.01 |

Note: Data expressed as means with standard deviation (mean±SD) . *p < 0.05 and **p < 0.01 compared with NC; #p < 0.05 and ##p < 0.01 compared with MCI group. Abbreviations: AD, Alzheimer’s disease; MCI, mild cognitive impairment; NC, normal control.

**Table 3 Predicted KEGG functional pathways differences at level 2**

| **KO functional categories** | | **AD**  **mean% (SD%)** | **MCI**  **mean% (SD%)** | **NC**  **mean% (SD%)** | ***P*-value** | | |
| --- | --- | --- | --- | --- | --- | --- | --- |
| **Level 1** | **Level 2** |  |  |  | **AD vs. MCI** | **AD vs. NC** | **MCI vs. NC** |
| Cellular Processes | Cell growth and death | 1.14 (0.20) | 1.29 (0.18) | 1.19 (0.17) | <0.01 | 0.021 | 0.016 |
| Cellular Processes | Cellular community - eukaryotes | 0.00 (0.00) | 0.00 (0.00) | 0.00 (0.00) | - | <0.01 | - |
| Cellular Processes | Cellular community - prokaryotes | 3.12 (0.67) | 2.67 (0.65) | 3.02 (0.60) | <0.01 | - | <0.01 |
| Cellular Processes | Transport and catabolism | 0.23 (0.10) | 0.29 (0.13) | 0.24 (0.11) | <0.01 | - | 0.020 |
| Environmental Information Processing | Membrane transport | 9.14 (1.70) | 7.94 (1.47) | 8.94 (1.60) | <0.01 | - | <0.01 |
| Environmental Information Processing | Signaling molecules and interaction | 0.00 (0.00) | 0.00 (0.00) | 0.00 (0.00) | - | 0.010 | - |
| Genetic Information Processing | Folding, sorting and degradation | 2.44 (0.23) | 2.58 (0.21) | 2.46 (0.21) | <0.01 | - | <0.01 |
| Genetic Information Processing | Information processing in viruses | 0.01 (0.01) | 0.01 (0.01) | 0.01 (0.01) | - | 0.017 | - |
| Genetic Information Processing | Transcription | 0.51 (0.14) | 0.50 (0.14) | 0.48 (0.09) | - | 0.012 | - |
| Human Diseases | Cardiovascular disease | 0.17 (0.02) | 0.17 (0.02) | 0.17 (0.02) | 0.042 | - | - |
| Human Diseases | Drug resistance, antimicrobial | 1.42 (0.27) | 1.48 (0.23) | 1.48 (0.23) | - | 0.035 | - |
| Human Diseases | Drug resistance, antineoplastic | 0.11 (0.02) | 0.11 (0.02) | 0.11 (0.02) | - | 0.018 | - |
| Human Diseases | Infectious disease, parasitic | 0.05 (0.03) | 0.04 (0.02) | 0.05 (0.02) | 0.039 | - | - |
| Human Diseases | Infectious disease, viral | 0.04 (0.12) | 0.03 (0.07) | 0.01 (0.00) | - | <0.01 | - |
| Human Diseases | Neurodegenerative disease | 0.13 (0.04) | 0.15 (0.03) | 0.12 (0.02) | <0.01 | - | <0.01 |
| Metabolism | Biosynthesis of other secondary metabolites | 0.79 (0.13) | 0.87 (0.14) | 0.82 (0.11) | <0.01 | - | 0.044 |
| Metabolism | Global and overview maps | 32.42 (1.00) | 33.14 (1.20) | 32.58 (0.99) | <0.01 | - | <0.01 |
| Metabolism | Glycan biosynthesis and metabolism | 2.53 (0.32) | 2.89 (0.43) | 2.59 (0.40) | <0.01 | - | <0.01 |
| Metabolism | Lipid metabolism | 1.68 (0.09) | 1.73 (0.12) | 1.71 (0.09) | 0.020 | <0.01 | - |
| Metabolism | Metabolism of cofactors and vitamins | 3.38 (0.26) | 3.62 (0.29) | 3.46 (0.21) | <0.01 | <0.01 | <0.01 |
| Metabolism | Metabolism of terpenoids and polyketides | 0.69 (0.06) | 0.71 (0.07) | 0.69 (0.06) | - | - | 0.031 |
| Organismal Systems | Development and regeneration | 0.01 (0.00) | 0.01 (0.00) | 0.01 (0.00) | 0.037 | 0.021 | - |
| Organismal Systems | Digestive system | 0.12 (0.04) | 0.15 (0.04) | 0.12 (0.04) | <0.01 | - | <0.01 |
| Organismal Systems | Endocrine system | 0.26 (0.05) | 0.28 (0.05) | 0.26 (0.04) | - | - | 0.048 |
| Organismal Systems | Immune system | 0.09 (0.02) | 0.10 (0.02) | 0.09 (0.02) | <0.01 | <0.01 | <0.01 |
| Organismal Systems | Nervous system | 0.06 (0.01) | 0.07 (0.01) | 0.06 (0.01) | <0.01 | <0.01 | <0.01 |

Note: Data expressed as mean% (SD%). Abbreviations: AD, Alzheimer’s disease; MCI, mild cognitive impairment; NC, normal control; KO, KEGG Ortholog.

**Table 4 Gut barrier dysfunction**

| **Biomarker** | **AD**  **(n=45)** | **MCI**  **(n=38)** | **NC**  **(n=35)** | ***P*-value** |
| --- | --- | --- | --- | --- |
| **DAO (U/L)** | 11.66±1.91* | 10.81±2.24 | 10.47±1.76 | 0.034 |
| **D-lactate (mg/L)** | 13.30±2.27** | 12.43±2.01* | 11.14±1.78 | <0.01 |
| **Endotoxin (U/L)** | 20.99±2.34**## | 18.21±2.23* | 17.01±1.37 | <0.01 |

Note: Data expressed as means with standard deviation (mean±SD) . *p < 0.05 and **p < 0.01 compared with NC; #p < 0.05 and ##p < 0.01 compared with MCI group. Abbreviations: AD, Alzheimer’s disease; MCI, mild cognitive impairment; NC, normal control.

# Supplementary Figures and Tables


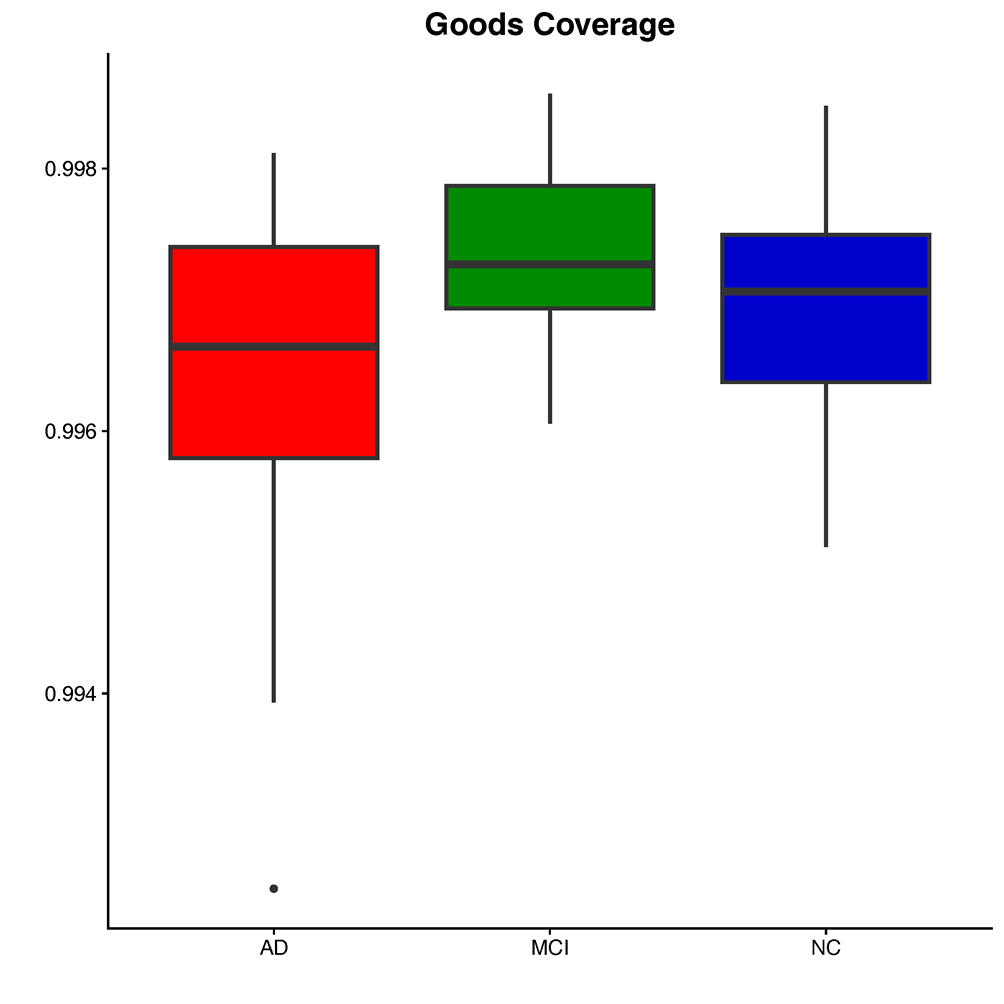


**Supplementary Figure S1 Goods Coverage analysis**


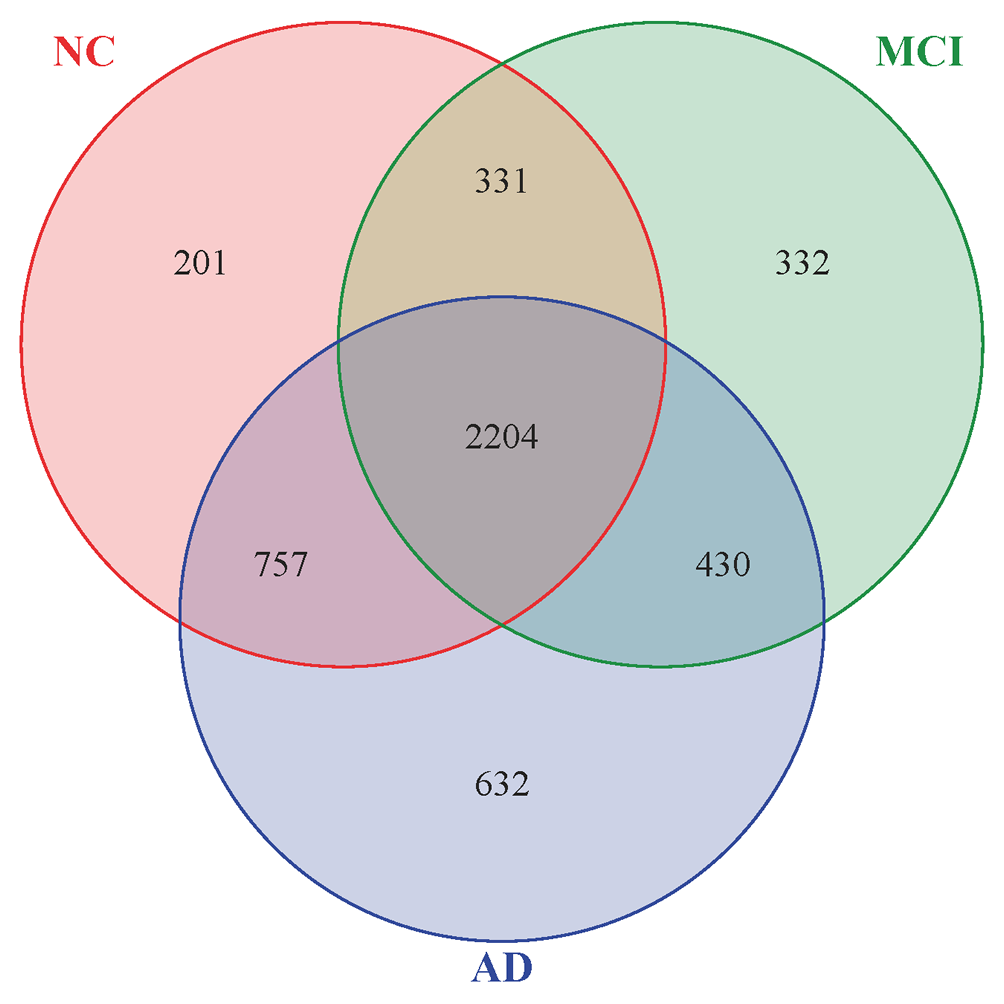


**Supplementary Figure S2 Venn diagram**

**
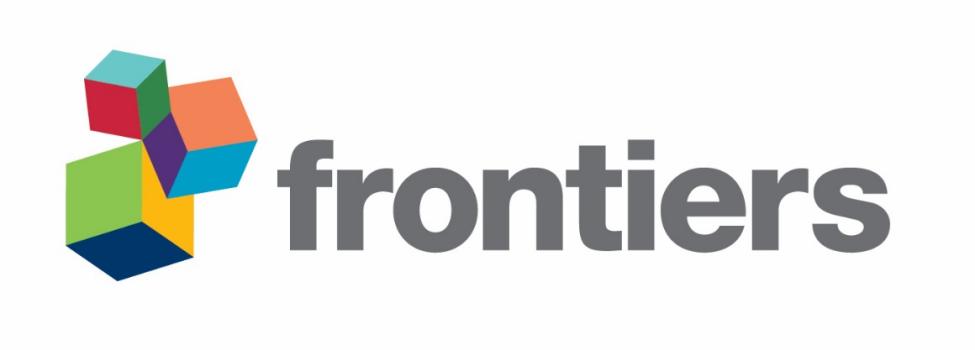
**
